# Natural Radiation in the Rocks, Soils, and Groundwater of Southern Florida with a Discussion on Potential Health Impacts

**DOI:** 10.3390/ijerph16101793

**Published:** 2019-05-21

**Authors:** Thomas M. Missimer, Christopher Teaf, Robert G. Maliva, Ashley Danley-Thomson, Douglas Covert, Michael Hegy

**Affiliations:** 1 Emergent Technologies Institute, U. A. Whitaker College of Engineering, Florida Gulf Coast University, 16301 Innovation Lane, Fort Myers, FL 33901, USA; rmaliva@fgcu.edu (R.G.M.); mhegy@fgcu.edu (M.H.); 2 Center for Biomedical and Toxicological Research, Florida State University, Tallahassee, FL 32310, USA; cteaf@fsu.edu; 3 WSP USA Inc., 1567 Hayley Lane, Suite 202, Fort Myers, FL 33907, USA; Robert.Maliva@wsp.com; 4 U. A. Whitaker College of Engineering, Department of Environmental and Civil Engineering, 10501 FGCU Boulevard South, Fort Myers, FL 33965-6565, USA; athomson@fgcu.edu; 5 Hazardous Substance and Waste Management Research, 2976 Wellington Circle West, Tallahassee, FL 32309, USA; dcovert@hswmr.com

**Keywords:** natural radioactivity, uranium, radium, radon, exposure, public health risk

## Abstract

Southern Florida is underlain by rocks and sediments that naturally contain radioactive isotopes. The primary origin of the radioactive isotopes is Miocene-aged phosphate deposits that can be enriched in uranium-238 and its daughter isotopes. Nodular phosphate containing radionuclides from the Miocene has been reworked into younger formations and is ubiquitous in southern Florida. When the nodular phosphate is exposed to groundwater with geochemical conditions favorable for its dissolution, uranium, radium, and radon may be released into the groundwater system. Uranium concentrations have been measured above the 30 µg/L drinking water standard at only one location in Lee County. Radium226/228 exceedances of the drinking water standard have been documented in numerous wells in Sarasota County. Indoor radon activities have exceeded the 4 piC/L guideline in five southern Florida counties. The exceedance of radioactivity standards in drinking water does not occur in municipal drinking water supplies, but rather only in some domestic self-supply wells. Health risks for exposure to radiation from domestic self-supply wells could be mitigated by testing of well water and, if necessary, switching to the use of a different aquifer or treatment process. While the risk of exposure to radon in indoor air in southern Florida is generally low, some areas are enriched in soil radon that migrates into structures, which could be addressed by improved ventilation.

## 1. Introduction

Natural radiation is a normal part of the environment that emanates from two main sources: cosmic radiation, which originates in outer space and passes through the atmosphere, and the decay of radionuclides (radioactive isotopes or radioisotopes) in the soil and rock. Radionuclides undergo spontaneous disintegration into daughter nuclides with an associated emission of ionizing radiation in the form of alpha and beta particles and gamma rays. Daughter nuclides may be either stable or may themselves be radionuclides which also undergo radioactive decay.

Essentially all rocks exhibit a low-level of natural radioactivity that is due to the decay of radionuclides that are typically present in minute quantities (e.g., parts per million). The natural radiation levels of soil and rock depend upon their concentrations of radionuclides and the specific activity of the radionuclides, which is defined as the number of decays per unit time per unit amount of substance. Specific activity is an inverse function of the half-life of the radionuclide and may be calculated based on numbers of decays of either the radionuclide or may also include decays of daughter radionuclides.

The primary sources of natural radioactivity in rock and soil are radionuclides of the elements uranium, thorium, and potassium (referred to as radioelements), specifically the uranium-238, thorium-232, and potassium-40 decay chains. The emitted radiation is due to both the decay of the parent radionuclides and their daughter radionuclides. The natural radiation of soil and rock depends upon mineralogical composition. Rocks composed of minerals with relatively high concentrations of uranium, thorium, and potassium have relatively high natural radioactivity. Soils typically reflect the radioelement concentrations of their parent rock. 

Radionuclides within rock and sediment may contribute to the radioactivity of groundwater only if they are dissolved or leached out of the source rocks and/or sediment and remain in solution (i.e., are not subsequently removed by precipitation or sorption reactions). The quantity of radionuclides that are released into the groundwater depends upon their concentration in mineral crystals or absorbed on sediments, and very importantly, the rate of dissolution, leaching, and desorption. 

**Figure 1:**
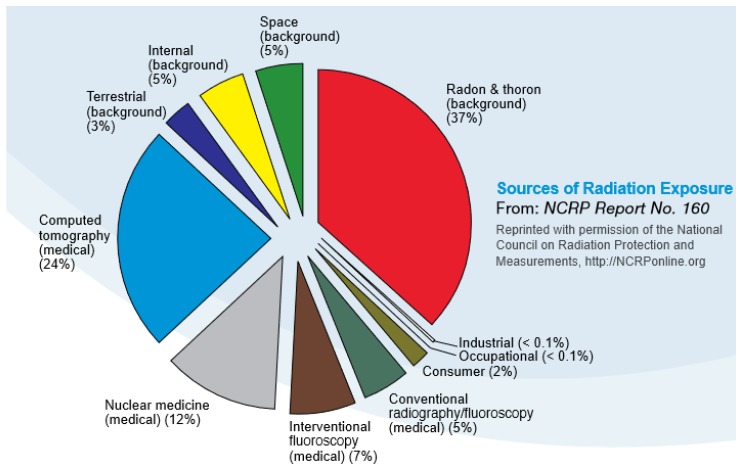
Average annual exposure to ionizing radiation of the United States population [3].

Human exposure to ionizing radiation is of interest to the population in general [1]. The U.S. Environmental Protection Agency (USEPA) estimates that the average human receives about 620 millirem of exposure to ionizing radiation per year [2]. The exposure profile occurs via three fundamental pathways, including natural influx from space (altitude dependent), local soils (radon and other radionuclides), and via health care procedures (e.g., CT scans, x-rays, therapeutic procedures). The relative degree of radiation exposure for an average person is shown in Figure 1. 

Southern Florida contains naturally occurring radioactive substances in rocks, soils, groundwater, and indoor air at various concentrations [4,5,6,7,8,9,10,11,12,13]. In addition, radioisotopes associated with atmospheric testing of nuclear weapons have accumulated in the soils and sediments of southern Florida and sometimes are mobilized into groundwater [14,15,16]. While radioactively in the soils and sediments of a carbonate platform is normally quite low, southern Florida may be an exception which should be of interest to the international community because background testing should be done even in areas where radioactively is not expected to occur. 

Human exposure to the naturally occurring radionuclides in southern Florida is generally limited to wind-blown organic soils, breathing of indoor radon gas, and in certain cases, the drinking of well water enriched in radionuclides. Radionuclides generally are removed in public drinking water systems via the treatment process which can be aeration (radon only), coagulation/filtration, lime-softening, ion exchange, activated alumina, or membrane processes [17]. Removal efficiencies range from 80 to 99% depending on the process used and the characteristics of the radionuclide [17]. Exposure to radionuclides in drinking water most likely comes from domestic self-supply wells for which there are no regulatory requirements to test for water quality. The concentrations of radioactive materials found in the southern Florida environment are herein documented with an assessment of potential health risks.

## 2. Uranium, Thorium, and Potassium Primary Decay Series

**Figure 2:**
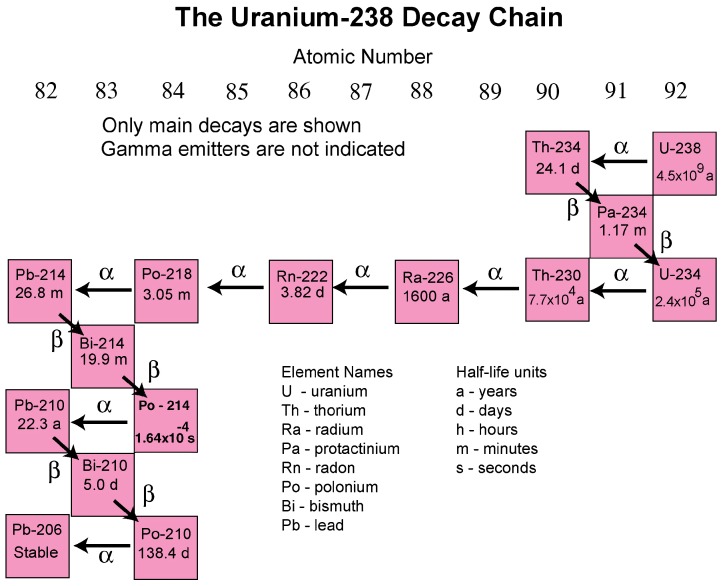
The uranium-238 decay chain (Source: U.S. Geological Survey [18]).

Approximately 99.3% of natural uranium occurs as the uranium-238 radionuclide, which has a half-life of about 4.5 billion years. The uranium-238 decay chain ends in the formation of the stable nuclide lead-206 (Figure 2) [18]. Daughter nuclides are radium-226 and radon-222 of which Radon-222 is the most common radon radionuclide. It forms by the alpha decay (emission of an alpha particle containing two neutrons and two protons) of radium-226. A primary health concern associated with uranium-238 and thorium-232 decay chains are the daughter products radon-222 and radon-220, respectively, because they can cause lung cancer upon sufficient exposure [19,20]. 

The amount of radon gas released to groundwater and the atmosphere are controlled by both the presence of minerals that contain uranium-238 and radium-226, and the radon emanation fraction of the mineral crystals present in rock, sediment, and materials derived from rock and sediment (i.e., some building materials). The radon emanation fraction is defined as the number of radon atoms released per number of radon atoms generated. Radon emanation fractions vary between minerals and are enhanced by increasing moisture contents, temperature, and specific surface area [21,22]. Radon emanation fractions of sediments (soil minerals) are much greater than those of the mineral in unweathered rock, which suggests that radon emanation fractions are dependent upon the degree of weathering [21]. Radon in the gas phase is mobile and may accumulate in confined spaces. Radon gas concentrations within dwellings are to a large degree controlled by home construction and characteristics (e.g., ventilation).

**Figure 3:**
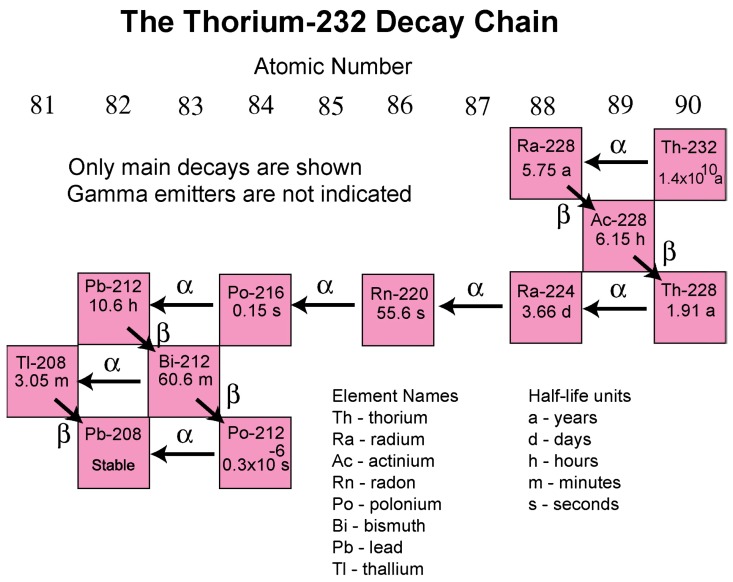
The thorium-232 decay chain (Source: U.S. Geological Survey [18]).

The thorium-232 decay chain is illustrated in Figure 3. The thorium-232 decay chain ends in the formation of the stable nuclide lead-208. Key daughter nuclides are radium-224 and radon-220. Radon-220 (which is referred to as thoron) forms by the alpha decay of radium-224 (Figure 3).

The potassium-40 decay chain is unusual in that the radionuclide decays into two main stable daughters. Approximately 89.3% of potassium-40 decays into calcium-40 with the emission of a beta particle. Approximately 10.7% of potassium-40 decays into argon-40 by electron capture with the emission of a gamma ray. Potassium is by far the more abundant form in rocks and sediments compared to uranium and thorium but potassium-40 constitutes only about 0.012% of potassium found in nature.

## 3. Radioactivity Units and Standards

Four main parameters are used in investigations of natural radiation and its potential human health considerations:

- concentration of radioelements
- radioactivity (decay rates)
- absorbed dose (level of human exposure to radiation)
- biologically effective dose—effect of radiation exposure on human tissues

Radioelement concentrations are expressed in standard concentration units, such as micrograms per liter (μg/L), milligrams per kilogram (mg/kg), or parts per million (ppm). Radioelement concentrations do not directly translate into radioactivity, which depends on the isotopic composition of the element.

Radioactivity, absorbed dose, and biologically effective dose are commonly expressed in traditional and SI (Système International) units, which are summarized in Table 1. Although utilization of SI units is preferred in the scientific literature, traditional units are still commonly used in the United States. Conversion factors of traditional and SI units are also provided in Table 1.

The units of radioactivity are curies (Ci) and becquerels (Bq). The curie is the originally accepted and still widely used unit, whereas the becquerel is the SI unit. The curie is now defined as 3.7 × 10^10^ radioactive decays per second. The commonly used picocurie (pCi) is equal to 1 × 10^−12^ curies or 0.037 decays per second. The Bq is defined as 1 radioactive decay per second. The radioactivity of materials is expressed with respect to a quantity of material (e.g., pCi/L, Bg/L, Bq/kg). For example, the radioactivity of water samples is commonly expressed in the United States in units of pCi per liter (pCi/L). The traditional and still widely used unit in the United States for absorbed radiation dose is the “rad,” which often is replaced by the SI unit, the “gray” (Gy), that is defined as the absorption of one joule of radiation energy by one kilogram of matter. One gray is equal to 100 rads. The traditional unit for the biologically effective dose of radiation is the “rem” (which stands for Roentgen equivalent in man). Biological exposure is commonly expressed in millirems (mrem), which are one-thousandth of a rem. The SI unit for biological exposure is the “sievert” (Sv), which is defined as the equivalent biological effect of the deposit of one joule.

## 4. U.S. EPA Standards and Criteria

The USEPA Maximum Contaminant Level (MCL, or primary drinking water standard) for uranium is 30 μg/L. Gross alpha and gross beta measurements are used as screening criteria and radionuclide-specific information cannot be obtained from the results. The main source of total alpha particles is radium-224 and radium-226, uranium, and radon. The current MCLs for radionuclides are given in USEPA [23]:

- Combined radium 226/228: 5 pCi/L
- Gross alpha: 15 pCi/L (not including radon and uranium)
- Beta emitters (e.g., strontium-90, tritium): 4 mrem/year

There is currently no USEPA groundwater or indoor air quality standard for radon. The USEPA [24] recommends that corrective actions be taken if radon levels in a home exceed 4 pCi/L and that radon levels less than 4 pCi/L may still pose a risk. In many cases, reductions below 4 pCi/L may be possible. The WHO [25] reference level for radon in indoor air is 100 Bq/m^3^ (2.7 pCi/L). Drinking water quality standards (MCLs) are set using risk analysis based on the increased risk of cancer and other diseases from a lifetime exposure. 

## 5. Treatment of Radon from Drinking Water

If combined radium-226/-228, (adjusted) gross alpha, uranium, or beta particle and photon radioactivity are present above standards or guidelines in the raw water before treatment, specific water treatment methods can be utilized to remove these radionuclides to levels below the MCLs. Reverse osmosis can be used to remove combined radium-226/-228, (adjusted) gross alpha, uranium, and beta particle and photon radioactivity, while ion exchange can be utilized to remove combined radium-226/-228, uranium, and beta particle and photon radioactivity. Lime softening can be used to reduce combined radium-226/-228 and uranium to concentrations below the MCLs. 

Water suppliers must monitor radionuclide levels in treated water routinely and notify customers within 30 days if a violation occurs. To decrease risk to public health, additional actions, like providing alternative water sources, may be implemented. The EPA requires that all community water systems must compose annually and deliver to customers a consumer confidence report (CCR), which is a water quality report that shows detected contaminants and compliance for various constituents, including radionuclides, if applicable. 

## 6. Occurrence of Radioactive Substances in Florida in Perspective

The occurrence of natural radioisotopes, particularly uranium and its daughter products, is commonly associated with organic-rich sediment marine and non-marine sediments in which uranium and other heavy metals may be concentrated [26,27,28,29,30]. Quite high uranium concentrations have been found in anoxic conditions in the presence of organic carbon. Mo et al. [28] found that uranium concentrations in sediments of the Pettaquamscott River, Rhode Island, increased proportionately with organic concentration, from 7 ppm at an organic content of 7%, to about 30 ppm at an organic carbon concentration of 14%. Many of the highest reported concentrations of uranium are found in black shales and phosphatic sediments [26,27,28,29,30]. Geochemically, uranium (IV) tends to adsorb onto accumulating organic matter during sedimentation in anoxic areas [31].

A large part of the Florida Peninsula is underlain by phosphatic sediments occurring within the Hawthorn Group of Late Oligocene to Early Pliocene age [32,33,34]. These phosphatic sediments can contain significant concentrations of uranium and its daughter radioactive isotopes [35,36,37,38,39,40,41,42,43]. In fact, at one time, investigations were conducted to assess whether these phosphorite deposits could be mined to produce uranium for various uses throughout the United States [41]. 

Analyses on the concentration of uranium, radium, and other radioactive isotopes have been conducted on the phosphate nodules, limestones, and groundwater in the Florida phosphate district [43]. A summary of the concentrations found is given in Table 2. Note that there are large variations in the measured activities due to primary lithological differences and perhaps subsequent diagenetic processes (e.g., dissolution, precipitation, and sorption/desorption) that may release or concentrate the radioisotopes. 

## 7. Radioisotopes in the Miocene to Recent Sediments and Groundwater of Southern Florida

### 7.1. Background Radioactivity

Phosphate sand grains and nodules originating in the Peace River Member of the Hawthorn Group in central Florida have been reworked and incorporated into younger geologic units in southern Florida [4,16,32,34,44,45], including a number of Pliocene and Pleistocene age units in Sarasota, Charlotte, Lee, Hendry, Martin, Palm Beach, Broward, and Dade counties. The reworked phosphate and some organic sediment accumulations, and associated anomalously uranium and radium-226 concentrations, have caused the occurrence of higher than background radioactivity levels in some areas, particularly in Sarasota and Lee counties and perhaps in Palm Beach County [45].

Several investigations in southeast Florida have documented the concentrations of various radioisotopes in shallow sediment, soils, and groundwater (Table 3).

A documented health threat caused by high gross alpha activities discovered in wells used to supply drinking water at a mobile home community and a school in Alva, Florida (Lee County) [5] led to several investigations of the occurrence of radioactive isotopes in the rocks and sediments in Lee County [7,10,12].

### 7.2. Investigations of Radioactivity in the Sediments of Lee County

**Figure 4:**
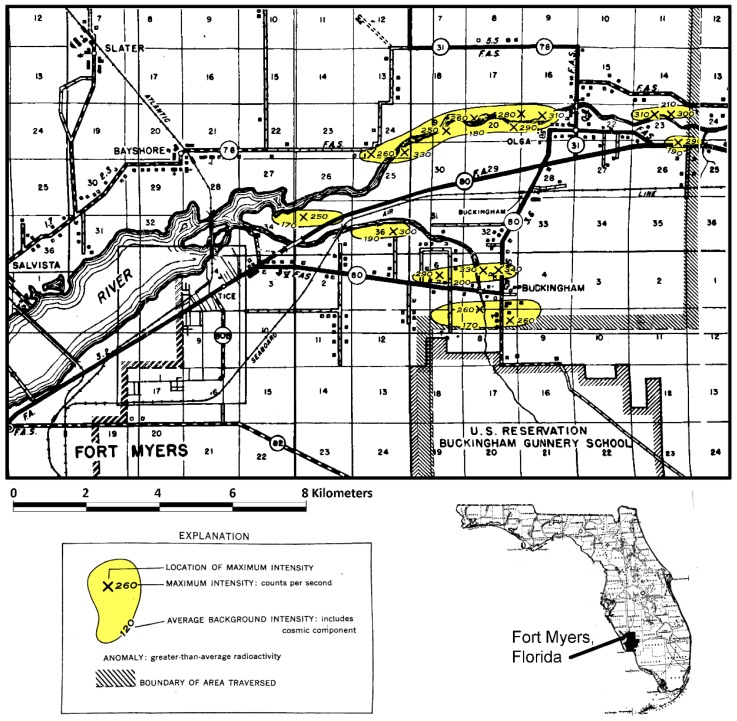
A portion of the aerial radioactivity survey conducted in southwest Florida in 1955 with the yellow areas being anomalies above background [46].

During World War II, the U.S. Army Air Force operated a gunnery school near Buckingham, Florida in Lee County. With the development of the first nuclear weapons, the U.S. Army performed some routine radioactivity background measurements near the Buckingham facility and found higher than background radiation activity values. In 1955, the U.S. Geological Survey in cooperation with the U.S. Atomic Energy Commission conducted an aerial radioactivity survey of parts of Lee, Charlotte, Hendry and Collier counties [46]. A series of “hot spot” areas were mapped in the northeastern part of Lee County (Figure 4). The background radioactivity was measured to be about 120 counts per second including the cosmic component. The discovered anomalies were above 170 counts per second and contained peak values of up to 330 counts per second. These anomalies were considered to represent deposits of radioactive material, but it could not be distinguished whether the radioactive source was uranium or thorium or some combination of both substances. During geologic mapping of Lee County conducted in the 1980s, it was found that these anomalies corresponded to the occurrence of the Buckingham Limestone Member of the Tamiami Formation and some of the Caloosahatchee Formation, which both contain reworked nodular phosphate [44,47,48]. 

Three investigations of the uranium concentrations in shallow geologic units were conducted in Lee County in the 1980s and 1990s [7,10,12]. Sediment samples were collected from four cores and analyzed from land surface to depths ranging from 140 to 235 m [7,12]. The uranium concentrations in the sediments were primarily located within macroscopic phosphate grains but also from clayey materials [36,43]. The phosphate sand and nodules typically contained uranium concentrations ranging from 30 to 100 ppm (mg/kg) in the southern extension of the Florida phosphate district and were higher in the land pebble part of the district being from 30 to 300 ppm with an average of 150 ppm [36]. 

**Figure 5:**
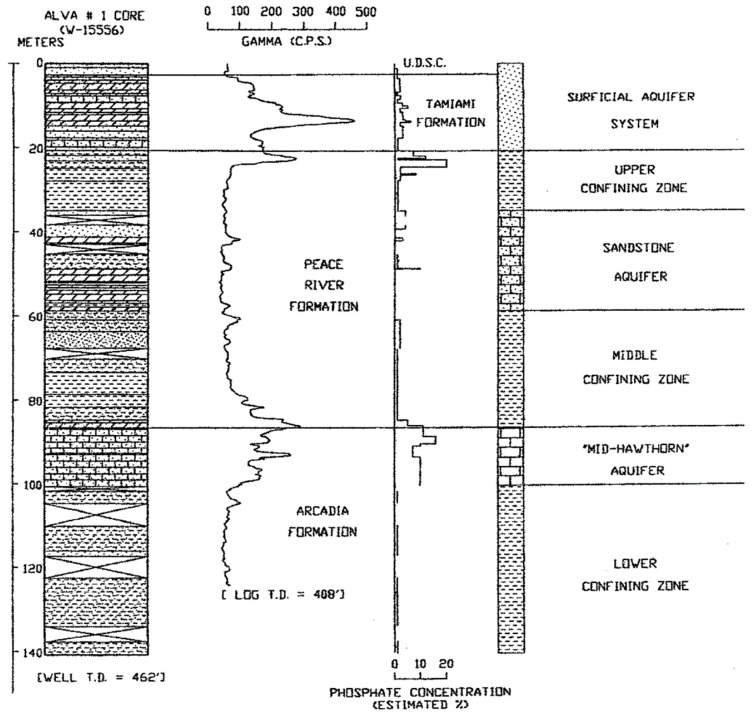
Relationship between the percentage of macroscopic nodular phosphate in the sediments and gamma-ray activity in the Alva core (from Green [12]).

**Figure 6:**
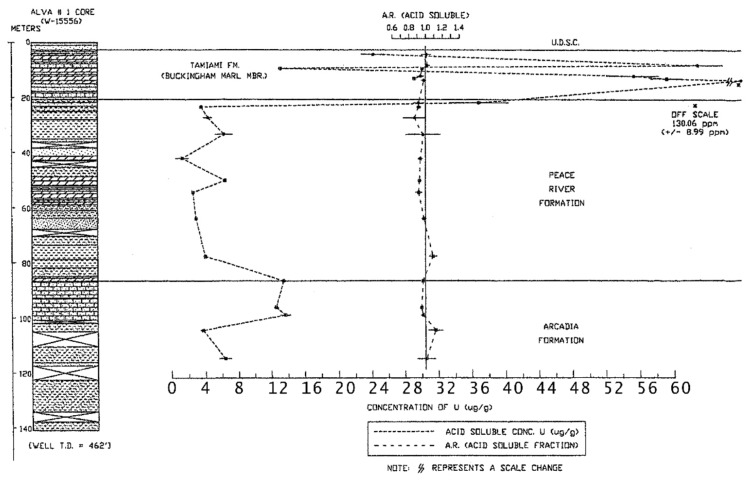
Variation in the concentration of uranium and the acid soluble fraction of uranium with depth in the Alva core (from Green [12]).

A direct association between gamma-ray activity measured in geophysical logs and the percentage of phosphate was found in all of the studied cores and there was a direct correlation with the occurrence of leachable uranium (Figure 5; [12]). However, Green [12] found that the gamma-ray activity in well logs was not dependent on the percentage of macroscopic phosphate nodules in the cores, but also on the clay-sized phosphate sediment and clays enriched in uranium. In addition, organic sediments also tended to be enriched in uranium and may also be present in the Buckingham Limestone member of the Tamiami Formation. This unit has measured concentrations of uranium ranging between about 15 to nearly 60 ppm (μg/g) (Figure 6; [12]). The total uranium concentration in the deeper geologic units, the Arcadia and Peace formations, ranged between 2 and 15 ppm. Anomalously high concentrations of uranium were found in the shallow sediments in the upper 50 m of the Alva core in Lee County, especially within the Buckingham Limestone Member of the Tamiami Formation which was also found in the aerial radioactivity survey [10,47]. Weinberg [10] measured uranium concentrations in phosphate nodules extracted from Buckingham Limestone at Alva to lie between about 440 and 729 ppm and concentrations in the bulk unsorted sediment were 45.4 ± 1.2 ppm at the top of the unit and 22.9 ± 0.4 ppm at the base of the unit [10]. In the Alva, Florida area, the high uranium concentrations in phosphate nodules also corresponded to high uranium and radium activities in groundwater [5,7]. Weinberg [10] suggested that the selective mobilization of the U(VI) fraction of the uranium present in the nodular phosphate in the Buckingham Limestone at Alva is the likely source of the uranium in the groundwater. 

## 8. Radiation in Groundwater

### 8.1. High Activities of Gross Alpha Radiation in Lee County, Florida Groundwater

Within one of the “hot spots” of natural radioactivity in Lee County, Pellicer and O’Connell [5] and the USEPA [38] reported exceptionally high activities of uranium and radium in groundwater, at 119 and 50 pCi/L, respectively. Outside of the Alva area in Lee County, the uranium activities were at 1 pCi/L or less [5]. Alpha emissions associated with uranium at activities above 50 pCi/L are considered to be very high in southern Florida, especially in consideration that the world-wide average concentration of uranium in groundwater is near 1 ppb with correspondingly low activities [39]. The uranium concentrations found were also unusual in that the activity ratio of ^234^U/^238^U were at or less than 1 [7]. Measurements were made of gross alpha activity in water from several wells, and they were reported to exceed the drinking water standard for the gross alpha activity of 15 pCi/L by a factor ranging from 3 to 40 times.

**Figure 7:**
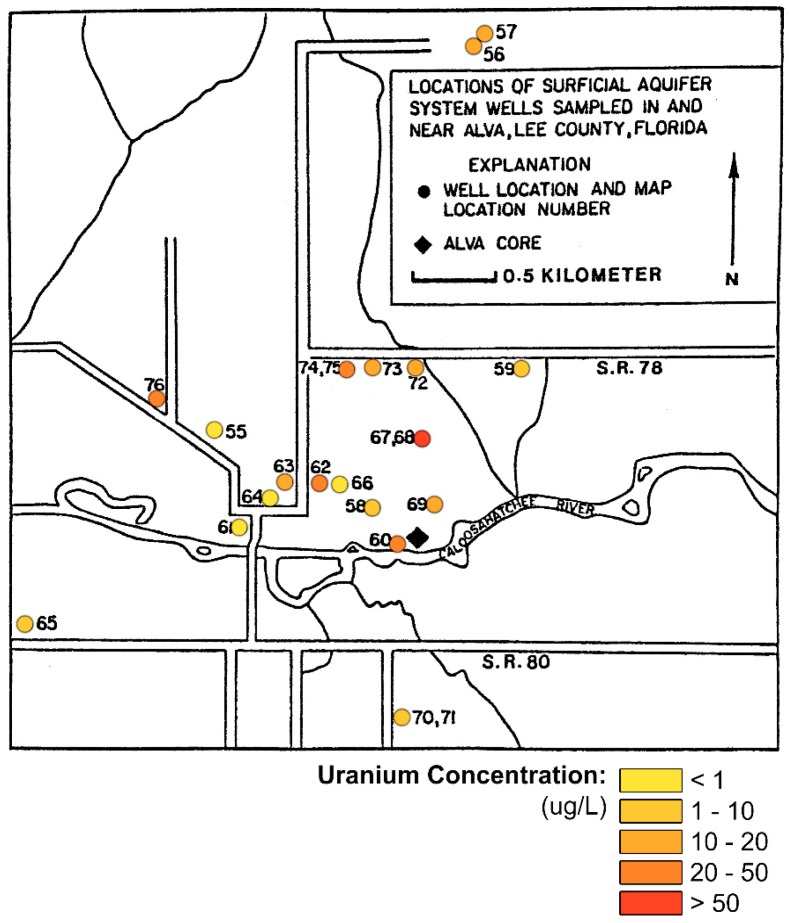
Uranium concentrations in the surficial aquifer at Alva, Florida (modified from Levine [7]).

In a comprehensive study of the groundwater radioactivity in the Alva area, Levine [7] measured the concentration of uranium in 22 wells that tap the shallow, unconfined aquifer (Figure 7). He found that the highest measured concentrations were 278 and 460 μg/L in the same supply wells at Oak Park Mobile Home Village (Alva, Florida, FL, USA) in which Pellicer and O’Connell [5] found very high gross alpha activities. Extreme variability was found in the uranium concentrations, from 0.2 to 49 μg/L, in shallow wells within a 1 km radius surrounding the Oak Park supply wells (wells 67 and 69 in Figure 7). In addition, the activity ratio of ^234^U/^238^U was nearly 1.0 in the majority of the water samples tested. This is an important issue because of the much higher specific activity of ^234^U at 0.33 μCi/g compared to ^238^U at 6220 μCi/g. Levine [7] also found that the leachable uranium was as high as 72 μg/L in the sediment samples.

### 8.2. Radium in Wells Located in Sarasota County, Florida

Background activities of radium were first reported in Central Florida by Kauffman and Bliss [38]. Radium-226 is a daughter product of the decay of ^238^U that commonly occurs in phosphatic sediments associated with the Miocene-age portion of the Hawthorn Group. Large quantities of nodular phosphate occur with the semi-confined aquifers located within the intermediate aquifer system of Sarasota County. Sutcliff and Miller [4] and Miller and Sutcliff [7] reported radium-226 activities ranging from below detection limits to 29 pCi/L. A summary of the data collected shows that 41.9% of the wells sampled contained water with no detection of radium-226 activity, 30.1% showed activity levels below the drinking water standard of 5 pCi/L (for combined ^226^Ra plus ^228^Ra), and 27.8% were above the drinking water standard (Table 4).

A much greater level of detail on radium-226 activity in the groundwater of Sarasota County was reported by Miller and Sutcliff [6] (Table 5). The well locations were defined by aquifer and showed that zones 1 to 3 of the intermediate aquifer system contain the highest natural activities of radium-226. The percentage of samples collected that exceed the drinking water standard of 5 pCi/L in Zones 1, 2, and 3 are 50%, 75.7%, and 40% respectively. Miller and Sutcliff [6] concluded that the radum-226 activities in the groundwater of Sarasota County were natural and correspond to water in the aquifers that has relatively high specific conductance and the occurrence of phosphate nodules in the sediments. Many homes in the area use wells tapping the various zones of the intermediate aquifer system for their water supplies.

### 8.3. Measured Radioisotopes in Southern Florida Groundwater and in Treated Drinking Water Supplies

Many municipal drinking water utilities in southern Florida report the activity of radioactive substances in their potable water supplies on an annual basis as part of the federally-mandated annual water quality report. A survey was conducted on the public drinking water supply systems and about half reported the activity of alpha emitters and the combined radium-226 and radium-228 activity. These values are reported in Table 6.

As shown in Table 6, the sources of raw water are primarily groundwater except the Lee County Utilities Olga Water Treatment Plant, which extracts water from the Caloosahatchee River. The upper part of the Floridan Aquifer System is used at the Lee County North Plant, and plants in Cape Coral, Sarasota County, Clewiston, and Belle Glade (Palm Beach County Lake Region Water Treatment Plant). The other utilities use shallow aquifers as their raw water supply source.

None of the utilities have reported any radioactive activities above drinking water standards. Four utilities have reported total uranium concentrations, all of which were well below the drinking water MCL of 30 μg/L. The values reported in Table 6 are the highest values measured during the 2017 reporting year. The highest gross alpha activity reported was at the Miami-Dade County South facility at 8.3 pCi/L, which is still below the standard for the gross alpha of 15 pCi/L. This value was associated with a relatively high concentration of uranium at 10 μg/L which was also well below the drinking water standard. No violations of drinking water standards were reported in the treated water.

## 9. Radon Production and Occurrence

### 9.1. Radon Production and Occurrence in Soils of Southern Florida as Indicated by Indoor Radon Activity

A statewide assessment of indoor radon activity was conducted by GEOMET for the Florida Phosphate Research Institute in 1987 [49]. The data compiled for southern Florida are shown in Table 7. Of the 10 counties in southern Florida, four had some indoor radon activities exceeding the standard (Lee, Hendry, Charlotte, and Dade counties). None of the measurements exceeded 8 pCi/L.

Another statewide assessment of indoor radon activity was published in 1993 by Otton [50]. The results for southern Florida are shown in Table 8. There are some significant differences between the two surveys, with the 1993 report showing a much greater number of radon activity exceedances above the 4 pCi/L guideline. Exceedances were found in Lee, Collier, Charlotte, Sarasota, and Dade counties, with Lee County reporting the highest radon activity values of up to 28.2 pCi/L. 

**Figure 8:**
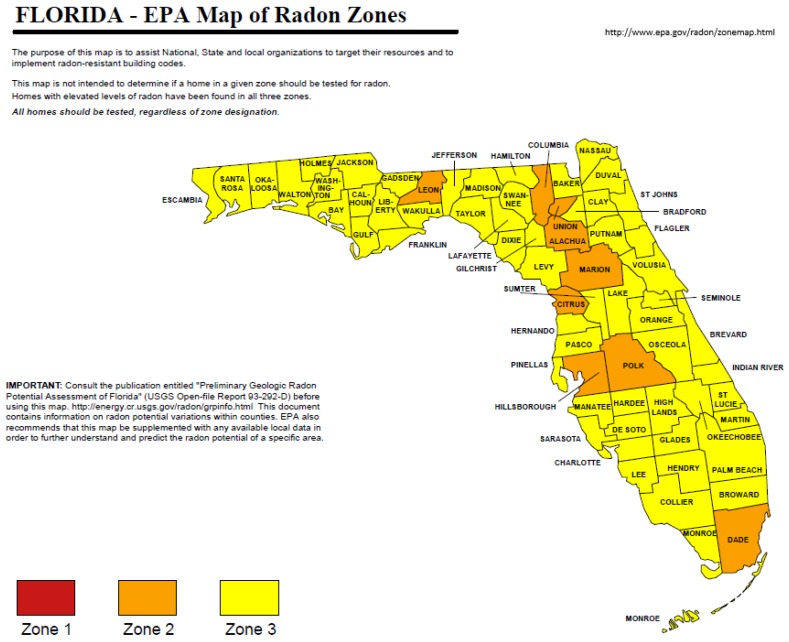
U.S. Environmental Protection Agency map of radon zones in Florida [51].

A statewide survey of radon activity risk was compiled as a map for Florida by USEPA from data acquired from a number of surveys (Figure 8; [50,51]). Only 9 of 67 counties in Florida showed a moderate risk for radon health risk. The yellow color denotes low risk, orange shows moderate risk, and red indicates high risk. In southern Florida, only Dade County was classified as a moderate risk with all other counties being classified as low risk. Given the results summarized in Table 8, it is interesting to note that Lee, Collier, Charlotte, and Sarasota counties were not among the moderate risk counties. It is likely that the radon survey data for Lee County may reflect in part the Alva, Florida data associated with the “hot spot” of radiation as previously described.

A groundwater quality study including radon in the Biscayne Aquifer in southeastern Florida was conducted by the U.S. Geological Survey between 1996 and 1998 [52]. The investigation included samples from public water supply wells and monitoring wells. There were 30 public supply wells sampled which showed minimum, median, and maximum radon activities of 202, 421, and 941 pCi/L respectively. The 32 monitoring wells tested showed the minimum, median, and maximum radon activities to be 230, 555, and 1800 pCi/L. Of the samples collected for radon-222, 86 percent exceeded what was at the time a proposed drinking water standard of 300 pCi/L [52].

**Figure 9:**
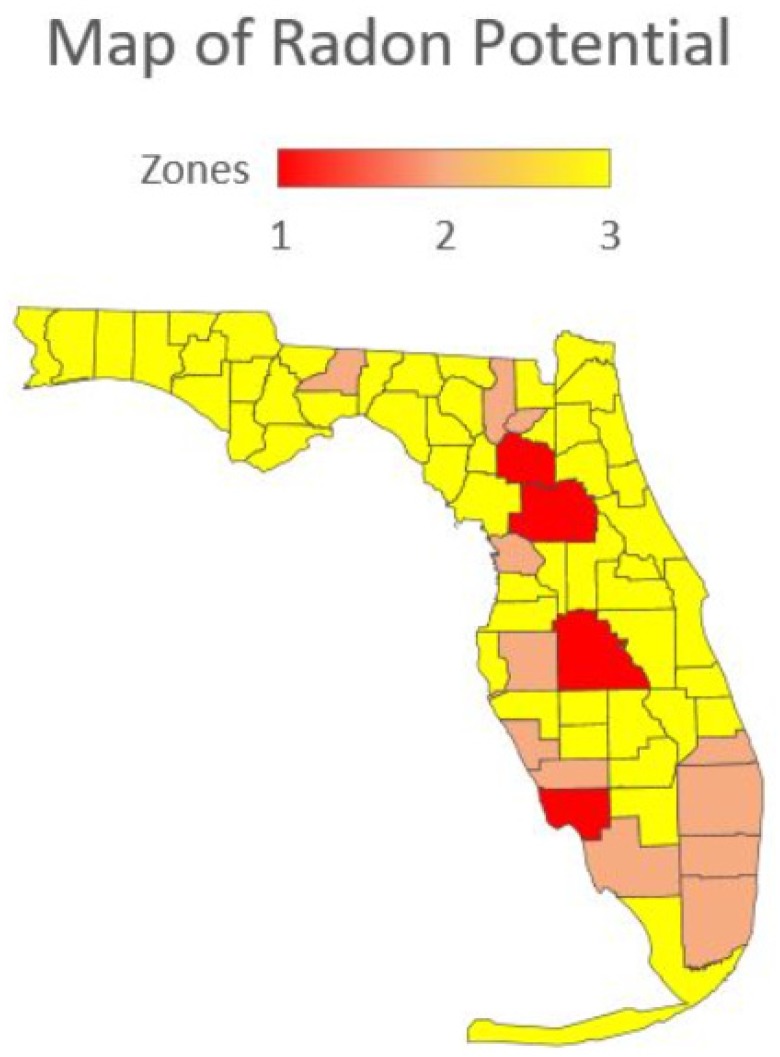
Radon potential zones from the Florida Department of Health [54].

The Florida Department of Health produced another guidance map for indoor radon potential in Florida [53] (Figure 9) that differs significantly from the USEPA map (Figure 8). This map shows Lee County with high potential, and Sarasota, Collier, Palm Beach, Broward, and Miami-Dade counties with a moderate potential status [53]. This map appears to follow the data collected from on-ground radon surveys to a closer degree.

### 9.2. Dade County Radon Anomaly

**Figure 10:**
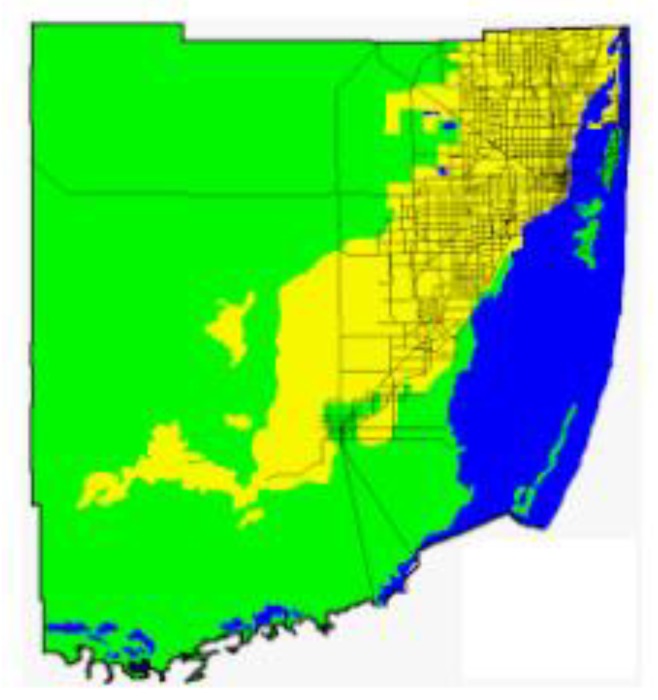
Recommended passive controls for radon in Miami-Dade County (Florida Department of Health [53]). Notes: Blue: Water; Green: Radon Controls Generally Unnecessary; Yellow: Passive Radon Controls Recommended.

A statewide radiation survey published in 1987 reported the results of indoor measurements of radon from 126 homes in Dade County [53]. These data showed elevated concentrations of radon in the south-central part of the county in the Homestead area. Moore and Gussow [9] documented the high radioactivity and associated concentrations of radon in the area. They collected samples of soil and rock (*n* = 88) and measured the radioactivity. Samples of the Miami Limestone yielded activities ranging from 0.17 to 0.73 pCi/g and the soils had activities from 0.13 to 8.69 pCi/g with a mean of 1.54 pCi/g [9]. The highest concentrations of radium were in the Rockdale soils which did not correlate to the earlier mapped highest aerial radiation measurements [54]. Otton and Asher-Bolinder [11] collected and analyzed samples of limestone, weathered limestone, clayey, iron-rich sands, peaty sands, and clean, well-sorted sands. The results of the analyses were as follows: (1) one sample of fresh Miami Limestone (radium = 1.1 pCi/g, emanating power = 0.05), (2) nine samples of weathered Miami Limestone (radium = 0.8–3.4 pCi/g, emanating power = 0.05–0.33), (3) 7 samples of clayey, iron-stained sand (radium = 1.9–11.3 pCi/g, emanating power = 0.49–0.86), and (4) peaty sand and clean sand (radium < 0.4 pCi/g, emanating power = undetectable). The authors also reported the results of a Florida Department of Health and Rehabilitative Services investigation which included 1,548 indoor radon measurements that showed a cluster of values at or above 4 pCi/g in south-central Dade County (Figure 10) and in the northeastern part of the county in multistory buildings with deep foundations. Otton and Asher-Bolinder [11] suggested that the source of the radon is a clayey “limonitic” horizon in the deep foundation building sites and that the uranium and radium enriched horizon was formed by a combination of limestone weathering and the influx of transported dust that is enriched in uranium and radium as documented by Rydell and Propero [55].

An area in southwestern Dade County, underlain by thin sandy soils covering shallow limestone bedrock, has equivalent uranium (eU) values as high as 3.5 ppm. Unusually high levels of radium are present in soils formed on the Pleistocene Key Largo Limestone and perhaps on other rock formations in certain areas of the Florida Keys and in southwestern Dade County. Areas of elevated eU and elevated indoor radon in Dade County are likely related to these unusual soils. These soils may be responsible for the modestly elevated eU in soils and for the elevated indoor radon levels, and they may extend into Collier County as well.

Because of the elevated radon activities in Miami-Dade County, the Florida Department of Health produced a map of Miami-Dade County showing areas where passive controls for indoor radon are recommended [53]. The recommended passive controls area is yellow on the map (Figure 10) and corresponds primarily to the occurrence of the “ridge area of the Miami Limestone” (oolite facies).

## 10. Discussion

### 10.1. Health Considerations Related to Radionuclides in the Environment

As thoroughly detailed previously, naturally occurring radionuclides are prevalent throughout southern Florida rocks, soils, and other environmental media. In this discussion section, these naturally occurring radionuclides are placed in perspective from a human exposure and health standpoint.

**Figure 11:**
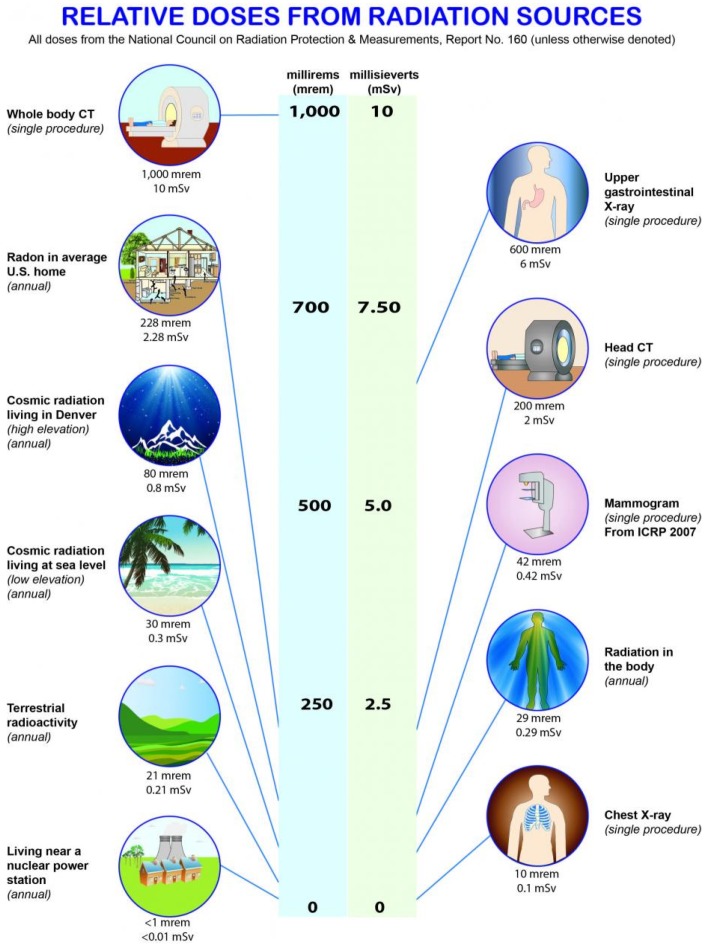
Relative doses from radiation sources (Source: NCRP Report 160 - Ionizing Radiation Exposure of the Population of the United States [66]).

Over the course of a year, the average American receives a radiation dose of approximately 620 millirem (620 mrem or 0.62 rem), a level that has been shown to not result in significant risk to humans [56]. Expressed in another common set of measurement units, 620 mrem/year is equivalent to 6.2 milliSieverts/year. That annual dose is about evenly split between natural background from terrestrial and cosmic sources, and humanmade background primarily from medical procedures. The major sources that contribute to the typical American’s annual radiation dose are presented in Figure 11. The human body has numerous intrinsic biological repair mechanisms that reduce the incidence of ill health from normal (background) levels of radiation to levels not normally discernible from other, non-radiation related causes [57].

Individual exposures largely will depend on the altitude of residence (relative closer proximity to cosmic radiation, coupled with less atmospheric attenuation), local soil types (sources of radon and thoron), and a number of nuclear medicine procedures or x-rays received. Lesser, but significant, sources of radiation exposure to the typical American include internal doses from food (e.g., bananas, Brazil nuts, lima beans, potatoes, red meat) and drinking water. Due to radiation present in the form of certain isotopes of potassium, radium, and others, all organic matter contains some small amount of radiation, and all water on Earth contains dissolved uranium and thorium. The typical annual dose from food and drinking water amounts to approximately 30 mrem [56].

Additionally, potential occupational exposure to radionuclides at varying levels can occur in a number of specific industries (e.g., airline crew members, mining), as well as medical institutions, educational and research establishments, and nuclear energy production facilities [58]. The dose from a cross-country (U.S.A.) airline trip is small (3.5 mrem), but frequent fliers may have high cumulative annual exposures [59]. As articulated by the International Atomic Energy Agency [60], “No human activity or practice is totally devoid of associated risks. Radiation should be viewed from the perspective that the benefit from it to mankind is less harmful than from many other agents.”

#### 10.1.1. Soils

Exposure to radiation in southern Florida soils typically is not a significant route of direct exposure, but the indirect contribution of soil-borne radionuclides to outdoor air, indoor air, and migration to groundwater can be significant in some instances. The primary route of exposure that is relevant to soil-borne radiation, particularly in southern Florida, is inhalation of radon and radon progeny in indoor air. Radon in outdoor air has not been shown to represent a human health risk. These radionuclides occur as a result of the natural breakdown of uranium in rock and soil, and also from the migration of uranium to groundwater [61]. The subsequent section on indoor air presents additional details on exposure to radon and radon progeny.

Issues have been raised over the years, and a number of studies have been conducted, regarding the contribution of phosphate mining and associated land reclamation to environmental radionuclide distribution. As noted previously, the radionuclides associated with phosphate deposits are naturally occurring by definition, though mining and processing operations can result in distributions that differ from the natural condition in some local soils. The Florida Department of Health (FDOH) is required by state law to assess pre-mining and post-mining conditions on phosphate lands. The annual reports of that FDOH program demonstrate that the phosphate areas can successfully and safely be reclaimed for a variety of uses, including agricultural, recreational, and residential [62,63,64]. Prior to 1975, the reclamation of phosphate mined lands was not required in Florida. For sites mined after 1975, the reclamation process presently is overseen by the Florida Department of Environmental Protection with regard to site requirements, monitoring, and financial responsibility [65]. 

#### 10.1.2. Groundwater

As with soil radionuclides, one area of interest regarding radionuclides in groundwater is the potential for impacts to indoor air from the use of impacted groundwater for domestic purposes (e.g., drinking water, bathing, and cooking). Based on a National Academy of Sciences report, the USEPA estimates that approximately 168 cancer deaths per year can be attributed to radon in drinking water. Of those deaths, 89% are from lung cancer caused by inhaling radon that was released into indoor air from water, and 11% are from stomach cancer caused by ingesting water containing radon [67]. As a point of comparison, that death rate is 4 to 5 times less than the number of annual fatalities in the U.S. from bicycle-related activities that are very common and generally perceived as safe and beneficial to health. Radon has not been shown to be a significant issue in Florida municipal drinking water supplies [68], though various forms of radioactivity have been reports in private drinking water wells. 

According to the USEPA [68] National Interim Primary Drinking Water Regulations, radium-226 is the most important of the naturally occurring radionuclides that may be present in public drinking water supplies. For public utilities that do not use groundwater as their source for drinking water, it has long been recognized that radium is not as much of a concern [69]. As previously detailed in Table 5 and Table 6, many south Florida water utilities, some of which rely on the Floridan Aquifer System for their water, report detectable levels of radium, but none exceed the MCL of 5 pCi/L. It is noted that all of these systems using the Floridan Aquifer System rely on reverse osmosis to desalt the raw water. 

#### 10.1.3. Other Media (Indoor Air)

Radon is regulated as a human carcinogen that reportedly results in more than 20,000 lung cancer deaths each year in America [70]; accounting for approximately 10% of U.S. annual lung cancer deaths [71]. In 2005, then current U.S. Surgeon General Richard Carmona released a national health advisory on radon [70] that warned in part, “Indoor radon is the second-leading cause of lung cancer in the United States and breathing it over prolonged periods can present a significant health risk to families all over the country.” He further reiterated, “It’s important to know that this threat is completely preventable. Radon can be detected with a simple test and fixed through well-established venting techniques.” It is significant to note that this advisory specifies indoor radon. The estimated risks from exposure to indoor radon are noticeably greater than those for outdoor radon, which is not recognized as a significant hazard. From 1999 National Research Council estimates, approximately 18,200 lung cancer deaths occurred from inhalation of radon progeny in indoor air and approximately 720 lung cancer deaths occurred from inhalation of radon progeny in outdoor air [71].

The USEPA estimates that the average indoor air radon level is 1.3 pCi/L, and the average outdoor level is 0.4 pCi/L [64]. As shown previously on Table 7 and Table 8, the average indoor air radon levels in counties of southern Florida are not significantly different from the 1.3 pCi/L national average, although the majority of the southern Florida readings were below 1 pCi/L. The radon protection maps and recommended control maps that were previously presented and discussed in Section 9, are components of the “Florida Standard for Passive Radon-Resistant New Residential Building Construction” (termed radon-resistant new construction or RRNC; [72]), as mandated in Part X, Chapter 553, Florida Statutes. The stated intent of the Florida Standard is to enable control of human exposure to indoor radon and its progeny [73]. The Florida Standard includes requirements for the sealing of concrete house slabs and below-grade walls for ventilation.

## 11. Conclusions

Southern Florida contains a significant amount of naturally occurring sources of radioactivity in soils, rocks, and groundwater. Concentrations of uranium in groundwater are generally below the drinking water standard of 30 µg/L except in a small area of Lee County at the town of Alva, where a 1988 evaluation reported exceedances of the standard in two wells. In a 1985 study, groundwater in Sarasota County within the Intermediate Aquifer System reported numerous exceedances of the drinking water activity standard for combined radium 226/228 in domestic self-supply wells. Indoor air radon measurements summarized in a 1993 report for five southern Florida counties showed exceedances of the recommended 4 piC/L guideline with up to about 10% of the measurements being at or above the guideline. 

Most of the naturally occurring radioactivity in the rocks, soils, and groundwater in southern Florida is associated with the occurrence of phosphate sand and nodules in sediments. These phosphatic sediments were originally deposited during Miocene time in the Hawthorn Formation to the north and were reworked and deposited into the younger rock formations. When phosphatic sediment is exposed to oxygenated groundwater, uranium and its daughter isotopes can be released into the groundwater. In all locations except Dade County, high activities of radon above the indoor air guideline are also associated with the local occurrence of phosphatic sediments. The Dade County radon occurrence is associated with marine sediments that contained organic matter with enriched uranium concentrations.

The potential health impacts of naturally occurring radioactive materials in groundwater in southern Florida is associated with domestic self-supply wells used for potable supply in geographically limited areas. No exceedances of radioactivity guidelines were found in municipal water supplies of southern Florida. 

The occurrence of radon in homes above the recommended guideline of 4 piC/L has been documented in five southern Florida counties. Individual home water supplies in identified hot spots should be tested to determine if groundwater levels may be contributing to indoor air levels. If the indoor activities are confirmed by repeated testing, these homes may be appropriate candidates for improved ventilation installations to reduce potential exposures.

## Figures and Tables

**Table 1:** Radiation units and conversion factors.

Parameter	Traditional Unit	SI Unit	Conversion Factor
Radioactivity	Curie (Ci)	Becquerel (Bq)	1 pCi = 0.037 Bq
Absorbed radioactivity	Rad	Gray (Gy)	1 Gy = 100 rads
Biologically effective dose	Rem	Sievert (Sv)	1 μSv = 0.1 mRem

**Table 2:** Background activities of selected radioisotopes in rock, nodular phosphate, and water in the central Florida phosphate area (from Burnett et al. [43]).

Isotope	Half-Life	Location Found	Activity
^231^Pa	3.276 × 10^4^ years	Phosphate rock, central FL	0.5–10.9 pCi/g
^210^Pb	22.3 years	Phosphate rock, central FL	11.9–161.4 pCi/g
^210^Pb	22.3 years	Unfiltered groundwater, central FL	0–7.70 pCi/L
^210^Po	138.376 days	Phosphate rock, central FL	11.0–134.5 pCi/g
^210^Po	138.376 days	Unfiltered groundwater, central FL	0–2570 pCi/L
^226^Ra	1600 years	Phosphate rock, central FL	13.3–165.2 pCi/g
^226^Ra	1600 years	Unfiltered groundwater, central FL	0–9.5 pCi/L
^222^Rn	3.8235 days	Unfiltered groundwater, central FL	6730–19,000 pCi/L
^230^Th	7.538 × 10^4^ years	Phosphate rock, central FL	13.6–198.8 pCi/g
^232^Th	1.4 × 10^10^ years	Phosphate rock, central FL	0.2–2.2 pCi/g
^234^U	24.1 days	Phosphate rock, central FL	13.3–250.8 pCi/g
^238^U	4.5 × 10^9^ years	Phosphate rock, central FL	12.8–252.5 pCi/g

**Table 3:** Radioactive isotopes known to occur within various geologic units and water of southern Florida.

Isotope	Half-Life	Location Found	Activity
^241^Am	432.2 years	Florida Bay sediments ^a^	0.0049–0.0131 pCi/g
^137^Cs	30.17 years	Florida Bay sediments ^a^	0–0.1847 pCi/g
^137^Cs	30.17 years	Everglades peat ^a^	8.34 × 10^−4^–0.073 pCi/g
^210^Pb	22.3 years	Florida Bay sediments ^a^	0.45–3.15 pCi/g
^210^Pb	22.3 years	Everglades peat ^a^	1.85 × 10^−4^–0.0285 pCi/g
^239 + 240^Pu	24,100 years, and 6563 years	Florida Bay sediments ^a^	0.00585–0.0333 pCi/g
^223^Ra	11.4 days	Everglades shallow water ^b^	0–0.0180 pCi/L
^223^Ra	11.4 days	Everglades peat ^b^	0.00022–0.0004099 pCi/g
^224^Ra	3.66 days	Everglades shallow water ^b^	0–0.00203 pCi/L
^224^Ra	3.66 days	Everglades peat ^b^	0.0095–0.01572 pCi/g
^222^Rn	3.8235 days	Shallow seawater near seawall (groundwater influence on seawater) ^c^	1.49–5.4 pCi/L
^226^Ra	1600 years	Groundwater (various aquifers) ^d^	<0.01–11 pCi/L
^238^U	4.5 × 10^9^ years	Groundwater (various aquifers) ^d^	<0.01–1.4 pCi/L

^a^ Robbins et al. [14], ^b^ Krest and Harvey [15], ^c^ Reich et al., [16], ^d^ Sutcliff & Miller, [4].

**Table 4:** Radium-226 activity measured in wells in Sarasota County, Florida (from Sutcliff and Miller [4]).

Activity Range for Radium-226 (in pCi/L)	Number of Samples	Percentage of Total
Not Detected	39	41.9
0–5	28	30.1
5–10	18	19.4
10–20	5	5.4
>20	3	3.2

**Table 5:** Radium-226 activity measured in wells tapping various defined aquifers in Sarasota County (from Miller and Sutcliff [6]).

Aquifer	Activity Range for Radium-226 (in pCi/L)	No. of Samples	Percentage of Total
Surficial	Not detected	0	0
	0–5	6	75
	5–10	2	25
	10–20	0	0
	>20	0	
Intermediate-Zone 1	Not detected	1	6.25
	0–5	7	43.75
	5–10	6	37.5
	10–20	1	6.25
	>20	1	6.25
Intermediate-Zone 2	Not detected	0	0
	0–5	8	24.2
	5–10	11	33.3
	10–20	10	30.3
	>20	4	12.1
Intermediate-Zone 3	Not detected	1	10
	0–5	5	50
	5–10	3	30
	10–20	1	10
	>20	0	0
Intermediate-Zone 4, Floridan Aquifer Systems-Zones 4 and 5	Not detected	0	0
	0–5	4	57.1
	5–10	3	42.9
	10–20	0	0
	>20	0	0

**Table 6:** Radioactivity in drinking water in southern Florida.

Utility	Year	Gross Alpha (pCi/L)	Radium 226+228 (pCi/L)	Uranium (μg/L)
Lee County Utilities-Pinewood	2017	6.70	2.50	-
Lee County Utilities-North	2017	-	2.26	-
Lee County Utilities-Olga (SW) ^1^	2017	-	1.10	-
City of Cape Coral-South	2017	9.14	1.78	-
City of Cape Coral-North	2017	3.82	1.05	-
Sarasota County	2017	3.02	1.05	-
City of LaBelle	2017	1.90	-	-
City of Clewiston	2017	-	0.06	-
Palm Beach County	2017	-	1.389	-
Palm Beach County-Belle Glade, Pahokee, South Bay	2017	-	-	0.019
Miami Dade County Main	2017	ND	0.3	1.20
Miami Dade County South	2017	8.3	0.9	10.0
Florida Keys Aqueduct Authority	2017	1.8	1.3	-
City of Homestead	2017	-	1.4	1.78

^1^ Surface water facility. Note that the utilities tap different aquifers ranging from the surficial system to the Floridan Aquifer System (treated using brackish water reverse osmosis).

**Table 7:** Indoor radon activity survey in southern Florida (from Nagada et al. [49]).

County	Number of Homes	Average (pCi/L)	Standard Deviation (pCi/L)	Maximum (pCi/L)	Percentage Homes (≥4 pCi/L)	Percentage Homes (≥8 pCi/L)	Percentage Homes (≥12 pCi/L)
Lee	144	1.1	1.0	5.8	2.1	-	-
Collier	43	0.7	0.5	3.1	-	-	-
Hendry	19	1.4	1.4	4.7	10.5	-	-
Charlotte	82	1.0	1.1	5.3	3.7	-	-
Sarasota	78	0.9	0.7	3.2	-	-	-
Glades	6	0.3	0.3	1.0	-	-	-
Palm Beach	68	0.5	0.4	1.6	-	-	-
Broward	43	0.3	0.4	1.8	-	-	-
Dade	55	1.0	1.1	4.7	3.6	-	-
Monroe	43	0.6	0.7	3.2	-	-	-

**Table 8:** Indoor radon gas activity in southern Florida (from Otton [50]).

County	Number of Homes	Average (pCi/L)	Standard Deviation (pCi/L)	Maximum (pCi/L)	Percentage Homes (≥4 pCi/L)	Percentage Homes (≥8 pCi/L)	Percentage Homes (≥12 pCi/L)
Lee	101	1.6	232	28.2	5.9	2.0	2.0
Collier	51	0.9	1.1	7.5	2.0	-	-
Hendry	13	0.6	0.7	2.2	-	-	-
Charlotte	39	1.7	1.4	6.1	10.3	-	-
Sarasota	135	0.7	0.8	5.6	1.5	-	-
Glades	6	0.4	0.4	1.1	-	-	-
Palm Beach	141	0.4	0.4	2.3	-	-	-
Broward	127	0.3	0.5	2.7	-	-	-
Dade	71	0.8	1.0	5.3	1.4	-	-
Monroe	9	0.2	0.0	0.2	-	-	-
